# The interaction between BSA and DOTAP at the air-buffer interface

**DOI:** 10.1038/s41598-017-18689-w

**Published:** 2018-01-10

**Authors:** Guoqing Xu, Changchun Hao, Lei Zhang, Runguang Sun

**Affiliations:** School of Physics and Information Technology, Shaanxi Normal University, Xi’an, 710062 China

## Abstract

In this article, the interaction between bovine serum albumin (BSA) and the cationic 1,2-dioleoyl-3-trimethylammonium-propane (DOTAP) at the air-buffer interface was investigated at different subphase’s pH values (pH = 3, 5 and 10). Surface pressure measurements (*π* − *A*) and penetration kinetics process (*π* − *t*) were carried out to reveal the interaction mechanism and the dynamical behavior. The data showed that *π* − *A* isotherms moved towards larger mean molecular area when the concentration of BSA ([BSA]) increased, the amount of BSA adsorbed onto DOTAP monolayer reached a threshold value at a [BSA] of 5 × 10^−8^ M, and BSA desorbed from the lipid monolayer as time goes by. The results revealed that the association of BSA with DOTAP at the air-buffer interface was affected by the subphase’s pH value. When pH = 10, the interaction mechanism between them was a combination of hydrophobic interaction and electrostatic attraction, so BSA molecules could be well separated and purified from complex mixtures. AFM images demonstrated that pH value and [BSA] could affect the morphology feature of DOTAP monolayer and the adsorption and desorption processes of BSA. So the study provides an important experimental basis and theoretical support for learning the interaction mechanism among biomolecules in separation and purification of biomolecules and biosensor.

## Introduction

In recent years, the research of the separation and purification of proteins has aroused extensive concern due to its increasing significance in diagnostics and therapeutics^1^. The separation and purification of proteins from complex mixtures can be implemented by several analytical methods, such as molecular recognition, capillary electrophoresis, molecular imprinting technology and so on^1–4^. Molecular recognition plays an essential role for the recognition of biomolecules, such as antibodies, enzymes and nucleic acids^5^. And the Langmuir-Blodgett (LB) technique is a comparatively simple way to investigate the mutual interaction mechanism between biomolecules, and transfer target biomolecules onto solid substrates with horizontal or vertical pulling methods. So the combination of molecular recognition and LB technique is a very efficient method for the separation and purification of proteins^2^. Therefore, the study of the interaction between protein and ligand provides an important experimental basis and theoretical support for learning the interaction mechanism among biomolecules in the fields of separation and purification of biomolecules and biosensor.

Bovine serum albumin (BSA) is one of the main proteins in bovine serum. It involves many biological functions, such as transporting metal ions, nutrients and drugs, maintaining osmotic pressure and buffering pH value^2,6^. BSA molecule contains six binding sites for long-chain fatty acids and several weak binding sites, all of them make BSA a preferential target for fatty acids^2^. And BSA has been widely used in health care and pharmaceutical applications^7,8^, which was because of its abundance, low cost, ready availability, unusual ligand-binding properties and its being homologous with human serum albumin (HSA)^9,10^. For example, BSA can be used as a blocking agent in the western blot, or a stabilizing agent in the reaction liquid^11–13^. So the separation and purification of BSA from complex mixtures are important for health care and pharmaceutical applications.

Compared with other conventional phospholipids, 1, 2-dioleoyl-3-trimethy- lammonium-propane (DOTAP) has a monocationic trimethylammonium head group and two unsaturated hydrocarbon chains^14^. Moreover, it is insensitive to pH and has a permanent cationic charge^15^. The unusual structure and positive electrical property make DOTAP apply in many fields. For example, DOTAP nanoliposomes containing antigens promote vaccines to elicit antitumor immunity^16^. And the phagocytosis can be efficiently enhanced by the electronic attraction between DOTAP liposomes and negatively charged molecules of target antigens^17^. Furthermore, DOTAP is also applied in non-viral vectorial gene therapy, transfection reagent and delivery system for drugs, peptides and DNA^18^. So DOTAP has a specific molecular affinity with other biomolecules.

In this work, DOTAP was used as a ligand for BSA. Experiments were performed to characterize the interaction between DOTAP and BSA at the air-buffer interface through the LB technique^19^ and atomic force microscopy (AFM)^20,21^, where the concentration of BSA ([BSA]) and subphase’s pH value were changed. According to the isoelectric point (IEP) of BSA (the IEP of BSA is from 4.6 to 5.1), three pH values (pH = 3, 5 and 10) were chosen. The net charge of BSA is positive at pH = 3, electrically neutral at pH = 5 and negative at pH = 10^22^, respectively. Surface pressure measurements were analyzed to learn the interaction mechanism between BSA and DOTAP, the surface compressibility of lipid monolayer and the dynamical behavior of BSA in the system. In addition, the morphology changes of DOTAP monolayer at different pHs can be observed from AFM images.

## Results and Discussion

### Molecular recognition between BSA and DOTAP molecules

**Figure 1:**
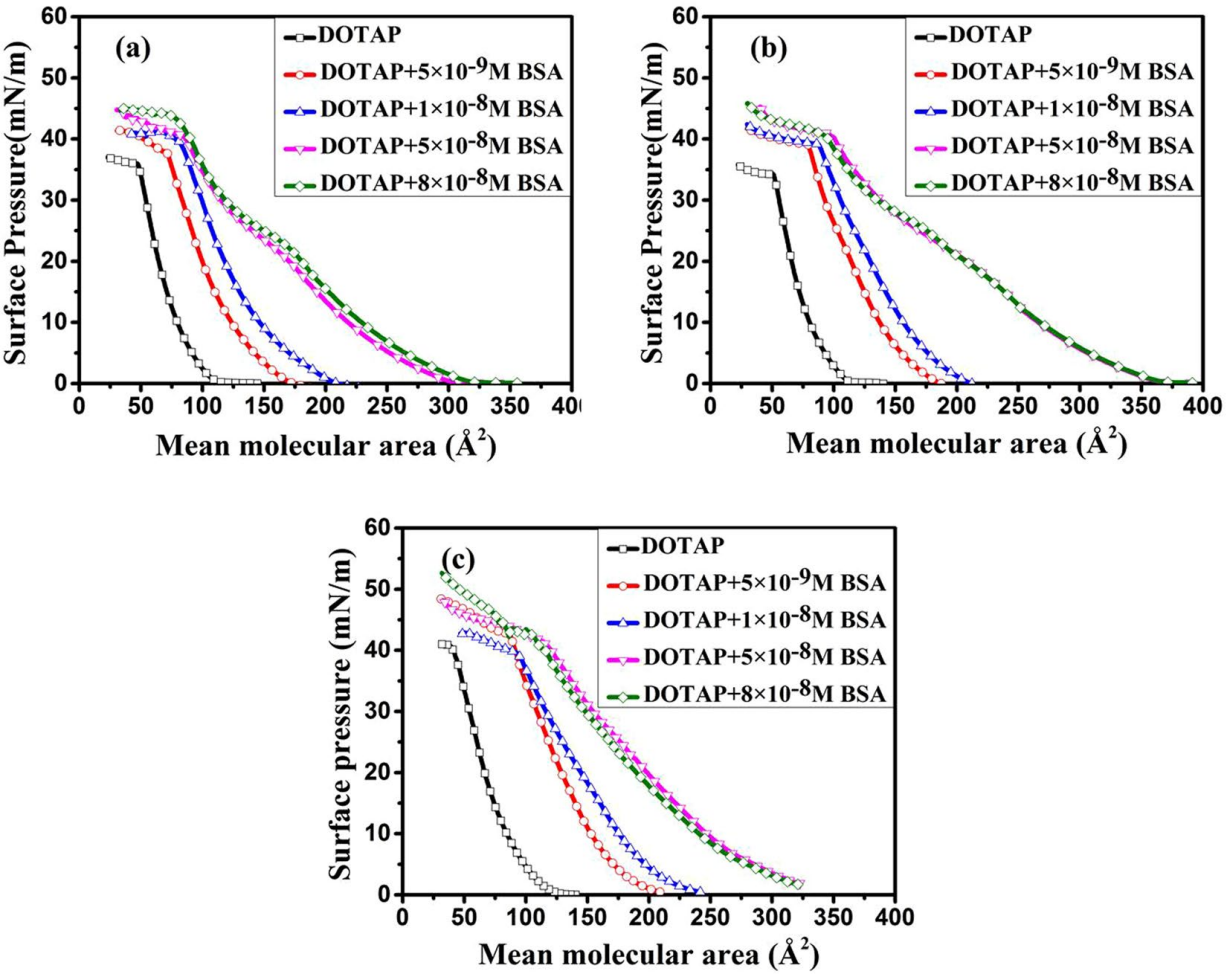
Surface pressure (*π*)-Mean molecular area (*A*) isotherms of DOTAP monolayer on the subphase with different amount of BSA (BSA concentration: 0(□); 5 × 10^−9^ M(○); 1 × 10^−8^ M(△); 5 × 10^−8^ M (▽); 8 × 10^−8^ M(◇)), pH = 3(a), 5(b) and 10(c).

In our work, surface pressure measurements were performed to study the association of BSA with DOTAP. The surface pressure-mean molecular area (*π* − *A*) isotherms of DOTAP monolayer on the subphase with different amount of BSA are shown in Fig. 1. As can be seen from Fig. 1, the isotherms of pure DOTAP monolayer showed continuous phase transition from gaseous phase to liquid condensed phase, and the collapse surface pressures (*π* coll) were between 35 mN/m and 43 mN/m. These results were consistent with the reported literature^23^. The *π* − *A* isotherms tended to shift to the larger mean molecular area with the increasing of [BSA]. This revealed that the interaction of DOTAP with BSA at the interface led to the expansion of the lipid monolayer. The *π*coll values of DOTAP monolayer were increased from 35.2 mN/m to 45.1 mN/m at pH = 3 Fig. 1(a), from 37 mN/m to 40 mN/m at pH = 5 Fig. 1(b), and from 41.1 mN/m to 53.8 mN/m at pH = 10 Fig. 1(c), with the addition of BSA. This indicated that the lateral movement and arrangement of lipid molecules were strongly affected by the adsorption of BSA. David Charbonneau *et al*.^24^ have investigated the interaction between DOTAP and HSA at pH = 7.4 through a combination of Fourier transform infrared (FTIR), circular dichroism (CD) and fluorescence spectroscopic method. They obtained that the binding of cationic DOTAP to HSA was mainly through hydrophobic interaction. In our work, electrostatic interaction existed between the charged molecules of BSA and the polar head of DOTAP at pH = 3 and 10, and non-electrostatic interaction existed at pH = 5, when BSA moved to the interface. If the binding of DOTAP to BSA was dominated by electrostatic interaction, the *π* − *A* isotherms of DOTAP monolayer would not shift to the larger mean molecular area with the addition of BSA at pH = 3. In addition, BSA is electrically neutral at pH = 5. So being homologous with HSA, the two unsaturated hydrocarbon chains of DOTAP may bind to the hydrophobic pockets of BSA at pH = 3 and 5, which resulted in the expansion. The early studies^2,25^ have proposed that the hydrophobic chains of N,N-dimethyl-PE and arachidic acid may bind to the hydrophobic pockets of BSA. However, when pH = 10, the adsorption of BSA onto DOTAP monolayer perhaps was dominated by a combination of electrostatic interaction and hydrophobic interaction. So the association of BSA with DOTAP at the air-buffer interface was affected by the subphase’s pH value.

**Figure 2:**
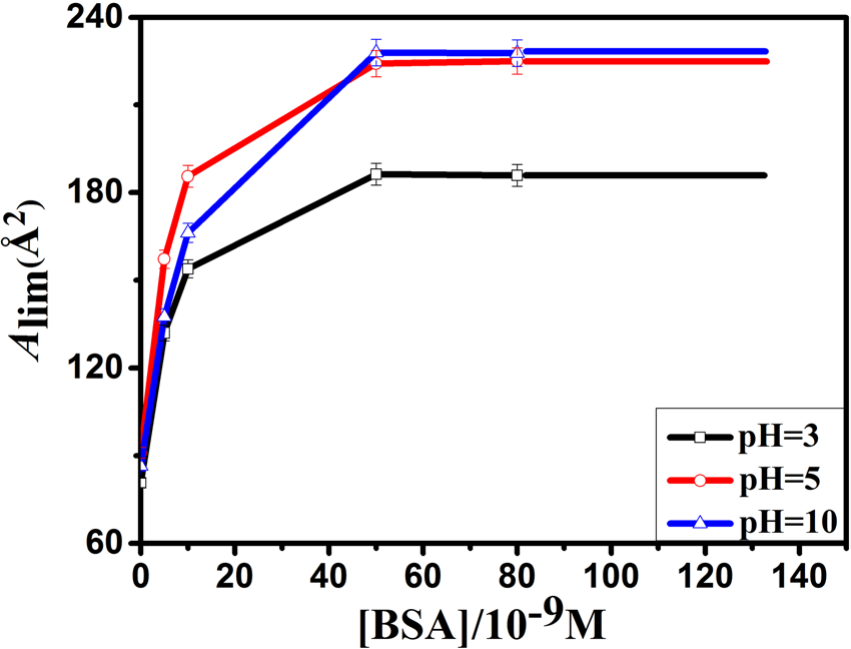
The limiting molecular area (*A*_lim_)-[BSA] curves of DOTAP monolayer on the subphase with different amount of BSA at pH = 3(□), 5(○) and 10(△).

The limiting molecular area (*A*lim) is the area occupied by one molecule in a highly compressed monolayer^26^, which can be used to character the change of DOTAP monolayer caused by BSA and pH value. It can be obtained by extending the steep linear part of the *π* − *A* isotherm to *π* = 0 mN/m^26,27^. The *A*lim-[BSA] curves of DOTAP monolayer are shown in Fig. 2. The *A*lim values of DOTAP monolayer were gradually increased with the increasing of [BSA], and remained approximately a constant value when [BSA] > 5 × 10^−8^ M. Moreover, as can be seen from Fig. 1, the isotherm of [BSA] = 8 × 10^−8^ M almost overlapped with that of [BSA] = 5 × 10^−8^ M at the same pH. This revealed that the amount of BSA adsorbed onto DOTAP monolayer reached a threshold value at a [BSA] of 5 × 10^−8^ M. A certain amount of DOTAP could absorb quantitative BSA at the air-buffer interface. So protein and lipid monolayer can be used in the biosensor field. Besides, Fig. 2 showed that the order of the constant *A*lim values at three pHs was *A*lim pH = 10 > *A*lim pH = 5 > *A*lim pH = 3. This demonstrated that the adsorption of BSA and pH value strongly affected the movement and arrangement of lipid molecules.

**Figure 3:**
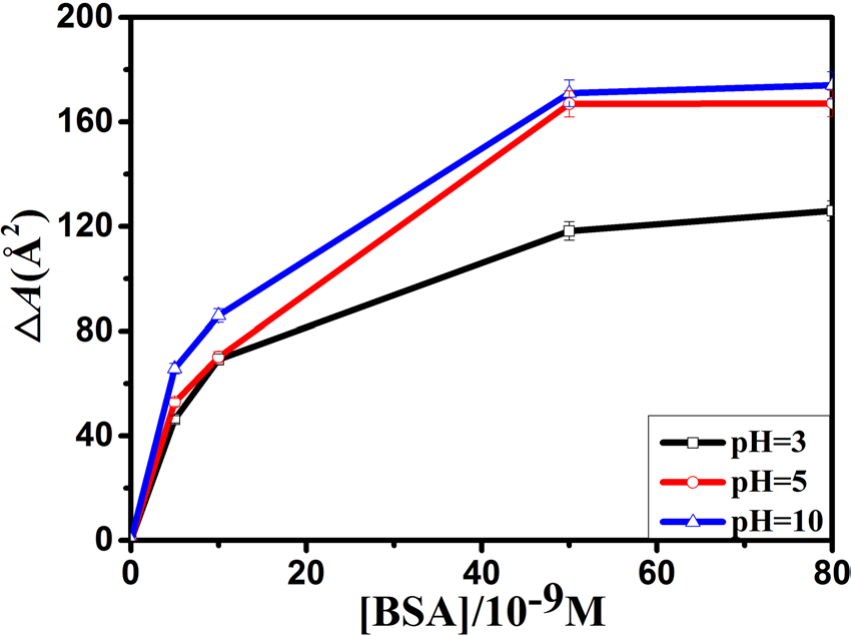
The difference value of mean molecular area (∆*A*)-[BSA] curves of DOTAP monolayer on the subphase with different amount of BSA at pH = 3(□), 5(○) and 10(△), *π* = 15 mN/m.

In order to character the change of *π* − *A* isotherms, the difference value of mean molecular area (Δ*A*) between mixed DOTAP-BSA monolayer and pure DOTAP monolayer at the surface pressure of 15 mN/m were calculated. The positive value means partial protein molecules adsorb onto lipid monolayer, while the negative value means aggregated protein molecules carry partial phospholipid molecules into subphase^28^. The Δ*A* − [BSA] curves obtained from *π* − *A* isotherms are shown in Fig. 3. As shown in Fig. 3, the Δ*A* values were positive at all pHs, and the order of Δ*A* values at the same [BSA] was Δ*A*pH = 10 > Δ*A*pH = 5 > Δ*A*pH = 3.

The order of *A*lim and ∆*A* values showed that BSA were much more readily adsorbed onto DOTAP monolayer at pH = 10. The reasons perhaps were that when pH = 3, BSA was positively charged and exposed most hydrophobic residues to the solution^2^. Besides, DOTAP is a positively charged lipid. So electrostatic repulsion and hydrophobic interaction existed between DOTAP and BSA at pH = 3. Electrostatic repulsion hindered BSA from moving to the interface. However, hydrophobic interaction was stronger than electrostatic repulsion, which led to the expansion of DOTAP monolayer. At pH = 5, BSA was electrically neutral, and in the most stable and compact form^2^. Thus, BSA was adsorbed to the interface mainly through hydrophobic interaction. At pH = 10, BSA was negatively charged and exposed less hydrophobic residues to the solution^2^. So the interaction between them was a combination of hydrophobic interaction and electrostatic attraction. As a result, the adsorption capacity of BSA onto DOTAP monolayer was the strongest at pH = 10. And the values of *A*lim and ∆*A* were the lowest at pH = 3, and the highest at pH = 10. These results indicated that BSA could be well separated and purified from complex mixtures at pH = 10.

### Compressibility analysis

The compressibility coefficient (${C}_{S}^{-1}$) is a useful parameter to quantify the surface compressibility of lipid monolayer and learn the details of phase transition behavior^29,30^. ${C}_{S}^{-1}$ can be calculated from *π* − *A* isotherms by the following equation:

1 $${C}_{S}^{-1}=-A{(\frac{\partial \pi }{\partial A})}_{T}$$

where *A* is the mean molecular area and *π* is the surface pressure.

According to the early studies by J. T. Davies *et al*.^31^, ${C}_{S}^{-1}\,$ can be used to quantify the physical state of lipid monolayer. The classification of the physical state of lipid monolayer is shown as follows: gas (G) phase (${C}_{S}^{-1}$ < 12.5 mN/m), liquid expansion (LE) phase (${C}_{S}^{-1}$: 12.5–50 mN/m), liquid (liquid expansion/liquid condensed coexistence (LE/LC)) phase (${C}_{S}^{-1}$: 50–100 mN/m), liquid condensed (LC) phase (${C}_{S}^{-1}$: 100–250 mN/m), and condensed (C) phase (${C}_{S}^{-1}$ > 250 mN/m)^26,31^. In general, the minima of ${C}_{S}^{-1}\,$ correspond to the phase transition point of lipid monolayer^32^.

**Figure 4:**
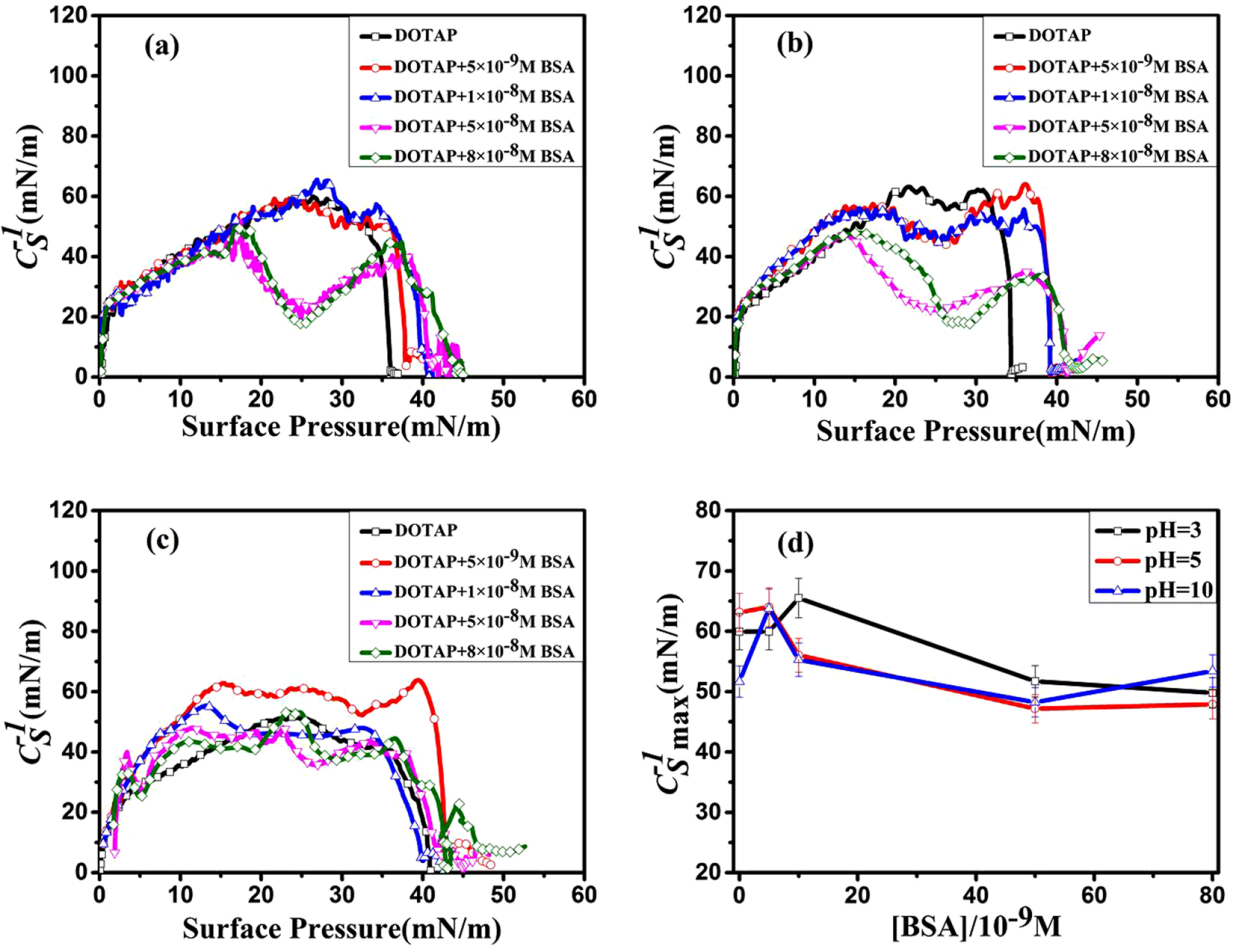
The Compressibility coefficient (${C}_{S}^{-1}$)-Surface pressure (*π*) curves of DOTAP monolayer on the subphase with different amount of BSA at pH = 3(a), 5(**b**) and 10(**c**) ([BSA] = 0(□); 5 × 10^−9^ M(○); 1 × 10^−8^ M(△); 5 × 10^−8^ M (▽); 8 × 10^−8^ M(◇)). (**d**): The variation of the maximum value of ${C}_{S}^{-1}$ (${C}_{S\,\max }^{-1}$) with [BSA] at pH = 3, 5 and 10.

The ${C}_{S}^{-1}$ − *π* curves are presented in Fig. 4. These curves revealed that with the addition of [BSA], phase transitions occurred at different surface pressures in the compression process. As shown in Fig. 4, pure DOTAP monolayer had no obvious phase transition points at the three pHs. At pH = 3, when [BSA]=5 × 10^−9^ M and 1 × 10^−8^ M, no obvious phase transition points were observed in the two curves. However, when [BSA] = 5 × 10^−8^ M and 8 × 10^−8^ M, two minima appeared at the same surface pressure of ~26 mN/m Fig. 4(a). It meant that the phase transition from LE to LE-LC phase occurred in the compression process with the increasing of [BSA]. At pH = 5, three minima were observed at the same surface pressure of ~25 mN/m with [BSA] increasing from 5 × 10^−9^ M to 5 × 10^−8^ M. In addition, It was also worth noting that when [BSA] = 8 × 10^−8^ M, a minimum was obtained at the surface pressure of ~29 mN/m Fig. 4(b). This revealed that the adsorption of BSA onto DOTAP monolayer caused the change of the phase transition behavior, and induced the phase transition points to move towards the higher surface pressure. In addition, we obtained that no obvious phase transition points were observed at the case of pH = 10 Fig. 4(c). In X. Wang *et al*.’ s work^33^, the presence of HSA changed the phase behavior of lipid monolayer, which was due to the adsorption and penetration of HSA into the phospholipid monolayer at the air-water interface.

In order to quantify the surface compressibility of lipid films, the change of ${C}_{S\,{\rm{\max }}}^{-1}$ (the maximum value of $\,{C}_{S}^{-1}$) with [BSA] is showed in Fig. 4(d). In general, the higher ${C}_{S}^{-1}\,$ value means the lipid monolayer is difficult to compress^26^. As shown in Fig. 4(d), ${C}_{S\,{\rm{\max }}}^{-1}$ values were first increased and then decreased with the increasing of [BSA] for all pHs. It meant the compression quality of DOTAP monolayer was initially decreased but finally increased with the increasing of the amount of [BSA] adsorbed onto lipid monolayer. The reasons were that at low [BSA], the adsorption of small amounts of BSA induced tight lipid monolayer, and the compression quality of DOTAP monolayer was decreased. When the amount of BSA exceeded the maximum adsorption capacity of DOTAP monolayer, partial DOTAP/BSA complexes were squeezed out from the interface in the compression process. As a result, some defects occurred in DOTAP monolayer, which caused looser lipid monolayer. This meant that the compression quality of DOTAP monolayer was increased with the increasing of [BSA]. J.H. Li, *et al*.^28^ have proposed that when the amount of protein exceeds the maximum adsorption capacity of lipid monolayer, partial phospholipid molecules will be carried into subphase, which promotes the compression quality of lipid monolayer. In addition, Fig. 4(d) showed that the compression quality of DOTAP monolayer at the same [BSA] was affected by pH value. The reason was that the interaction between BSA and DOTAP was changed with the variation of pH value. This was consistent with the analysis results obtained from *π* − *A* isotherms.

### Penetration kinetics at air-buffer interface

The *π - t* curves of DOTAP monolayer spreading on the subphase with the absence and presence of BSA ([BSA] = 0, 1 × 10^−8^ M) were obtained at constant monolayer areas after attaining the surface pressure of 15 mN/m. The penetration kinetics of BSA was studied by monitoring the reduction of *π* with time at different pHs. The desorption process of BSA followed a pseudo first order kinetics. And the first-order rate constant (*κ*) was calculated to learn the desorption process of BSA. It can be obtained from the equation (2)^34^.

2 $$\kappa =\frac{2.303}{t}\,\mathrm{log}\,\frac{({\pi }_{t}-{\pi }_{f})}{({\pi }_{i}-{\pi }_{f})}$$

where *π*_t_, *π*_*i*_ and *π*_*f*_ are the surface pressure of monolayer at time *t*, initial and final, respectively. *κ* is the first-order rate constant.

**Figure 5:**
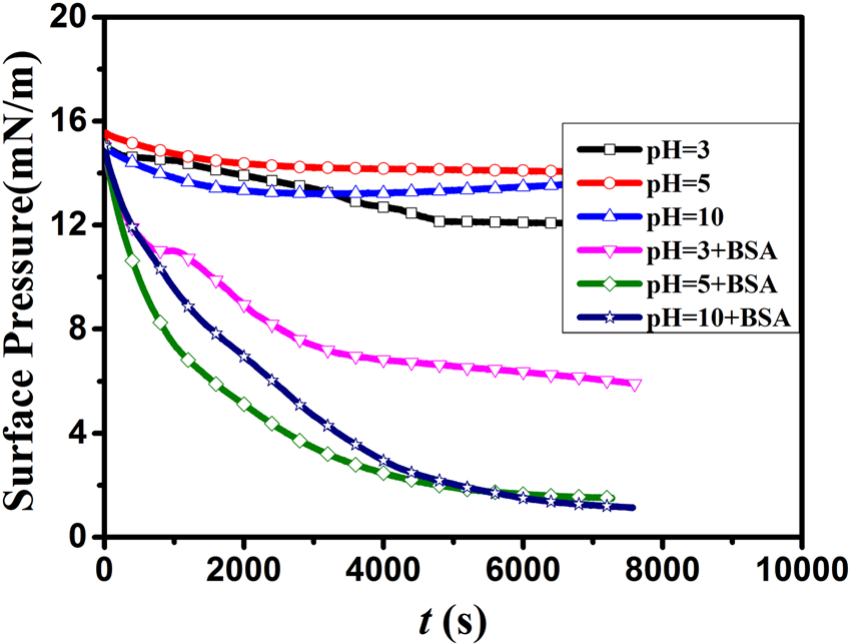
Surface pressure (*π*)-*t* of DOTAP monolayer on pure PBS subphase (pH = 3(□), 5(○) and 10(△) and containing BSA (1 × 10^−8^ M) subphase (pH = 3(▽), 5(◇) and 10(☆).

The *π* − *t* curves of DOTAP monolayer at the initial surface pressure of 15 mN/m are shown in Fig. 5 (pH = 3, 5 and 10, [BSA] = 0 and 1 × 10^−8^ M). The surface pressure will decrease or increase until the equilibrium value (*π*e) is reached. The difference value of *π*e (∆*π*) was calculated to character the change of *π*e (∆*π* = *π*_e2_ − *π*_e1_). The positive value means BSA exists onto lipid monolayer, while the negative value means no BSA exists and the number of phospholipid molecules decreases at the air/water interface. The *π*e, ∆*π* and *κ* values of DOTAP monolayer ([BSA] = 0, 1 × 10^−8^M) at different pHs are shown in Table 1.

**Table 1:** The equilibrium pressures (*π*_e_) and kinetic parameters (*κ*) of DOTAP monoalyer at different subphase pH ([BSA] = 0, 1 × 10^−8^M).

pH	*π*e_1_ (mN/m)	*π*e_2_ (mN/m)	∆*π* (mN/m)	*κ* × 10^4^ s^−1^	
	DOTAP	DOTAP-BSA		DOTAP	DOTAP-BSA
3	12.1	6	−6.1	6.843	6.248
5	14.0	1.5	−12.5	9.849	8.481
10	13.6	1.1	−12.5	16.300	7.377

The calculation of the κ value was based on the data of *π* − *t* curves from 0 to 5000 s and 0 to 7000 s for pure DOTAP monolayer and mixed DOTAP-BSA monolayer ([BSA] = 1 × 10^−8^ M), respectively.

As can be seen from Fig. 5, the surface pressure of DOTAP monolayer was decreased to an equilibrium value as time goes by (up to *t* ≈ 5000 s and 7000 s for pure DOTAP monolayer and mixed DOTAP-BSA monolayer, respectively.). This revealed that desorption process occurred at the three pHs. Table 1 showed that the *π*e values of pure DOTAP monolayer were 12.1 mN/m (at pH = 3), 14.0 mN/m (at pH = 5) and 13.6 mN/m (at pH = 10), respectively. In addition, the *π*e values of DOTAP monolayer were obviously changed with the addition of BSA. When [BSA] = 1 × 10^−8^ M, the *π*e values were nearly 1.5 mN/m (at pH = 5) and 1.1 mN/m (at pH = 10), respectively. While the *π*e value was nearly 6 mN/m at pH = 3. These indicated that more molecules existed at the interface at pH = 3. The reason was that the combination of hydrophobic interaction and electrostatic repulsion made less BSA molecules adsorb onto the lipid monolayer. So a small quantity of DOTAP was taken away from the interface in the desorption process of BSA. In the end, more molecules existed at the interface. However, at pH = 5 and 10, the interaction between DOTAP and BSA was stronger than that at pH = 3. What resulted in smaller *π*e value was that a large quantity of DOTAP was carried into subphase by BSA. Moreover, the ∆*π* values were negative at the three pHs. It also meant partial DOTAP molecules were carried into subphase in the desorption process. The results could also be obtained from AFM images. So the behavior of BSA in the system undergoes four important stages: diffusion, initial adsorption, desorption progress and equilibrium state rearrangement.

**Figure 6:**
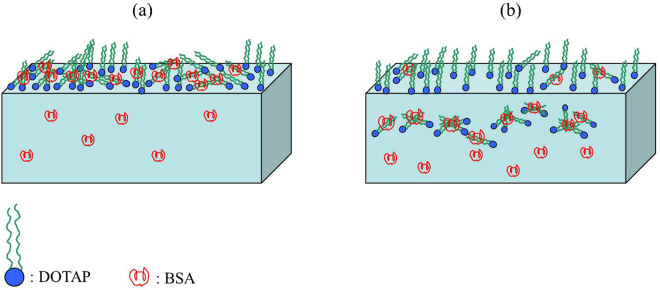
The model system of the adsorption process (**a**) and desorption process (**b**) of BSA in the system.

As can be seen from Table 1, *κ*_pH=3_ = 6.843, *κ*_pH=5_ = 9.849 and *κ*_pH=10_ = 16.300 for pure DOTAP monolayer, respectively. The *κ* values were increased with the increasing of pH value, which meant the molecular rearrangement of lipid monolayer at pH = 10 was much more intense than that at pH = 3 and 5. In addition, we could also obtain that *κ*_pH=3_ = 6.248, *κ*_pH=5_ = 8.481 and *κ*_pH=10_ = 7.377 for mixed DOTAP-BSA monolayer, respectively. At the same pH, the *κ* value was decreased with the addition of BSA. K. Maiti *et al*.^34^ have proposed that the desorption rate of BSA was decreased with the increasing of [BSA], which was because the inter-protein molecular interaction slowed down the desorption rate. The order of *κ* values of mixed DOTAP-BSA monolayer was *κ*_pH=3_ < *κ*_pH=10_ < *κ*_pH=5_. At pH = 3, the *κ* value was minimum. In addition, it is worthy to note that, the *κ* value at pH = 10 was lower than that at pH = 5. The reasons perhaps were that in the desorption process of BSA, a small amount of BSA was adsorbed onto DOTAP monolayer. While, the intensity of the interaction between DOTAP and BSA at pH = 10 was stronger than that at pH = 5, so BSA was easily adsorbed onto DOTAP monolayer at pH = 10 in the desorption process. As a result, the *κ* value at pH = 10 was lower than that at pH = 5. The model system of the adsorption and desorption processes of BSA is shown in Fig. 6. Firstly, BSA was absorbed onto DOTAP monolayer, and the two kinds of molecules formed complex compounds Fig. 6(a). Then, partial complex compounds desorbed from the interface, and less phospholipid molecules were left at the interface Fig. 6(b).

### AFM observation

**Figure 7:**
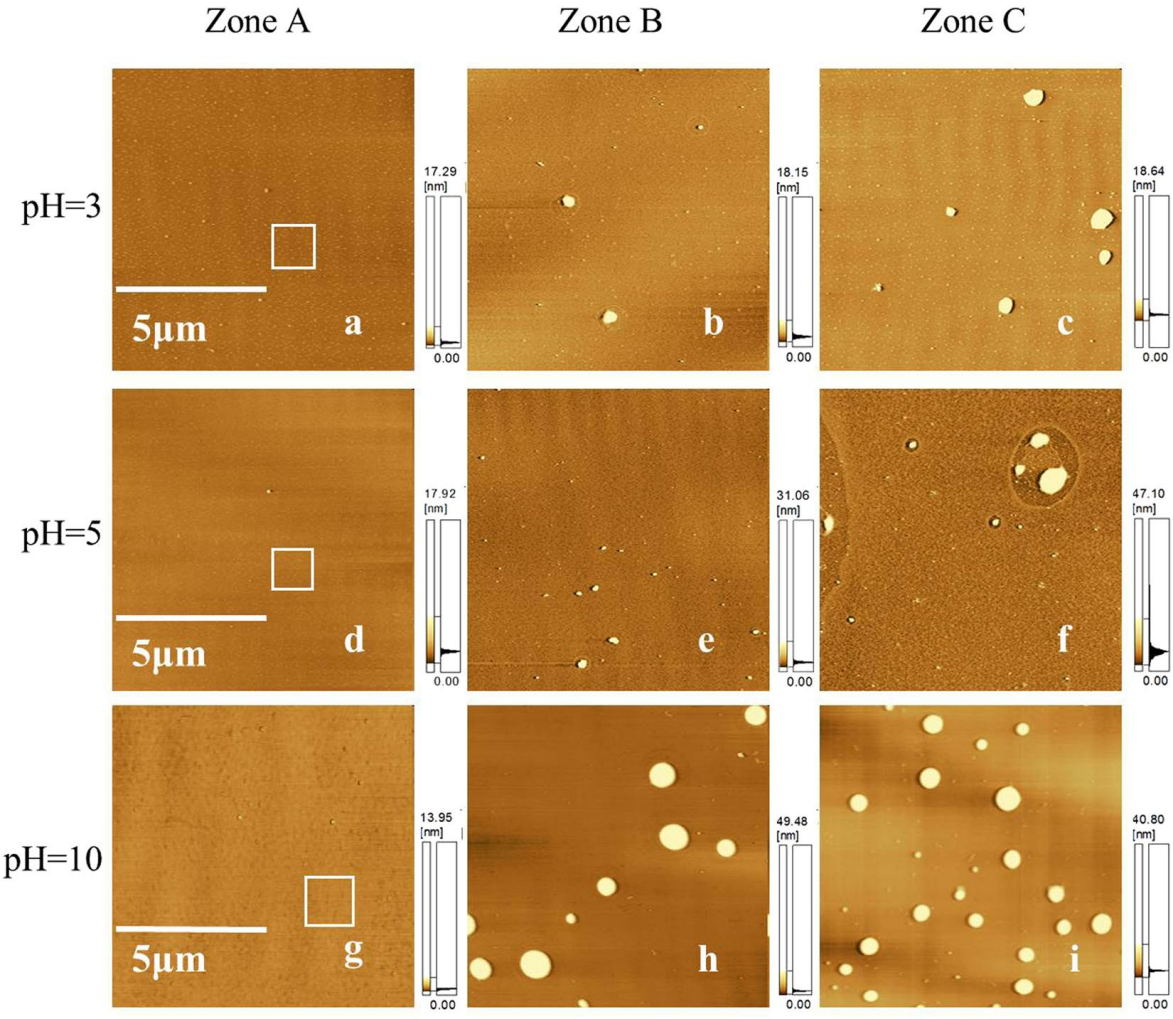
AFM images of DOTAP monolayer on the subphase with different amount of BSA at the surface pressure of 25 mN/m at pH = 3, 5 and 10. ([BSA] = 0 (Zone A); [BSA] = 1 × 10^−8^ M (Zone B); [BSA] = 5 × 10^−8^ M(Zone C)). Scanning range: 10 μm × 10 μm.

**Figure 8:**
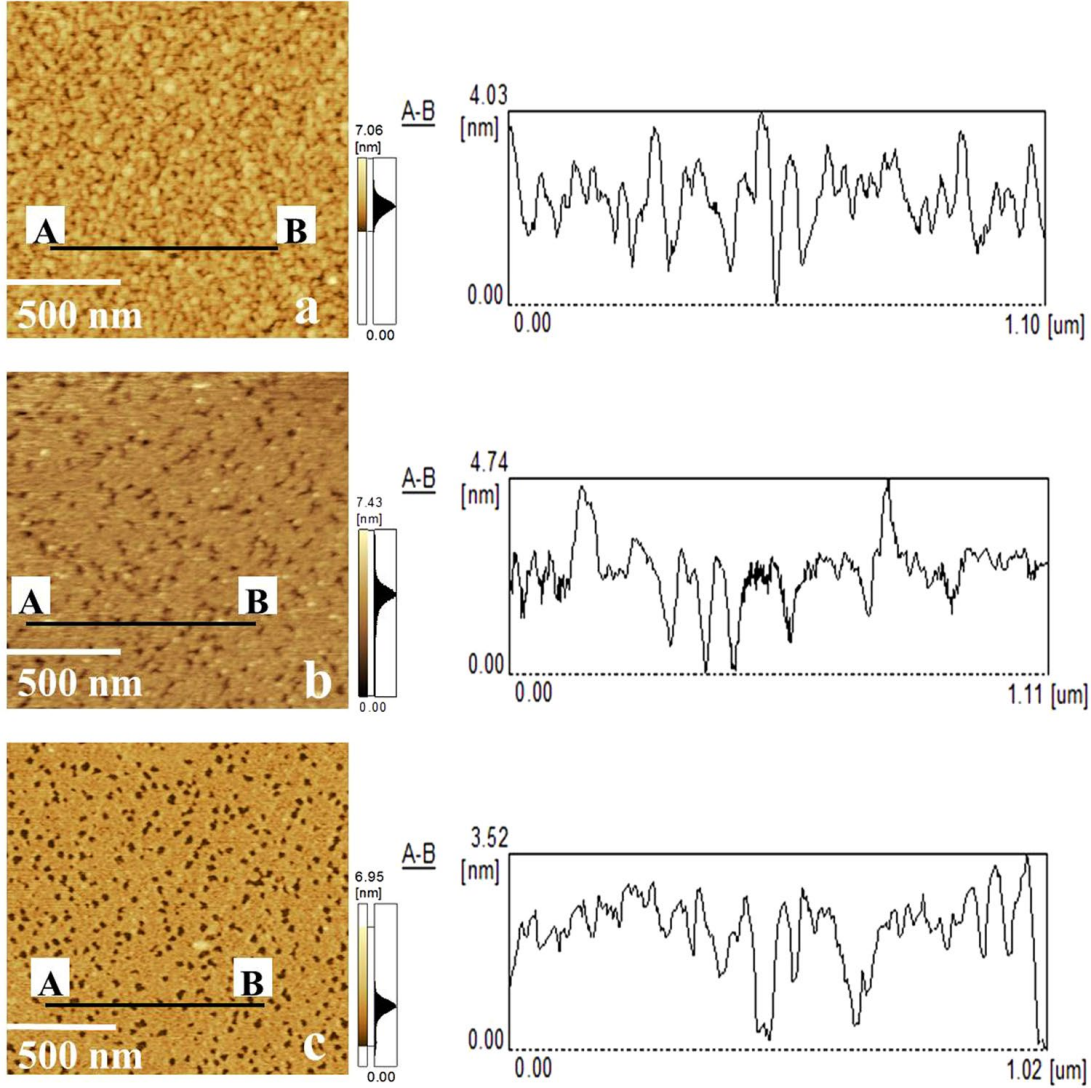
AFM images and the corresponding profiles of AB lines of the tagged areas of Fig. 7(a,d) and (g) (pH = 3(a), 5(**b**) and 10(**c**)). Scanning range: 1.5 μm × 1.5 μm.

At different pHs, the morphologies of DOTAP monolayer on the subphase with different amount of BSA ([BSA] = 0 M, 1 × 10^−8^ M and 5 × 10^−8^ M) at the surface pressure of 25 mN/m are shown in Fig. 7. AFM measurements of pure DOTAP monolayer were performed in small range (1.5 μm × 1.5 μm), which were corresponding to the tagged areas of Fig. 7, are shown in Fig. 8. In addition, the corresponding profiles of AB lines in AFM images are also shown in Fig. 8. The profiles showed that the thicknesses of pure DOTAP monolayer were about 4.03 nm (at pH = 3), 4.74 nm (at pH = 5) and 3.52 nm (at pH = 10), respectively. This revealed that DOTAP tended to form a thicker film at pH = 5. Besides, the root mean squared roughness (RMS) of the observed domains were 0.744 nm (at pH = 3), 0.574 nm (at pH = 5) and 0.722 nm (at pH = 10), respectively. The roughness of pure DOTAP monolayer was the highest at pH = 3. It meant the variation of pH value could affect the roughness of pure DOTAP monolayer. With the increasing of [BSA], more BSA molecules were seen in observed domains of mixed DOTAP-BSA monolayer at the same pH [as can be seen from Fig. 7(b,c,e,f,h) and (i). However, it was obvious to see that at the same [BSA], the amount of BSA in the observed domains was increased with the increasing of pH [as can be seen from Fig. 7(b,c,e,f,h) and (i). It also demonstrated that the strength of the interaction between DOTAP and BSA was the strongest at pH = 10. These results were consistent with the results obtained from surface pressure isotherms. Figures 7 and 8 showed that BSA molecules were adsorbed onto DOTAP monolayer in the beginning. The subphase’s pH value had an effect on the adsorption of BSA.

**Figure 9:**
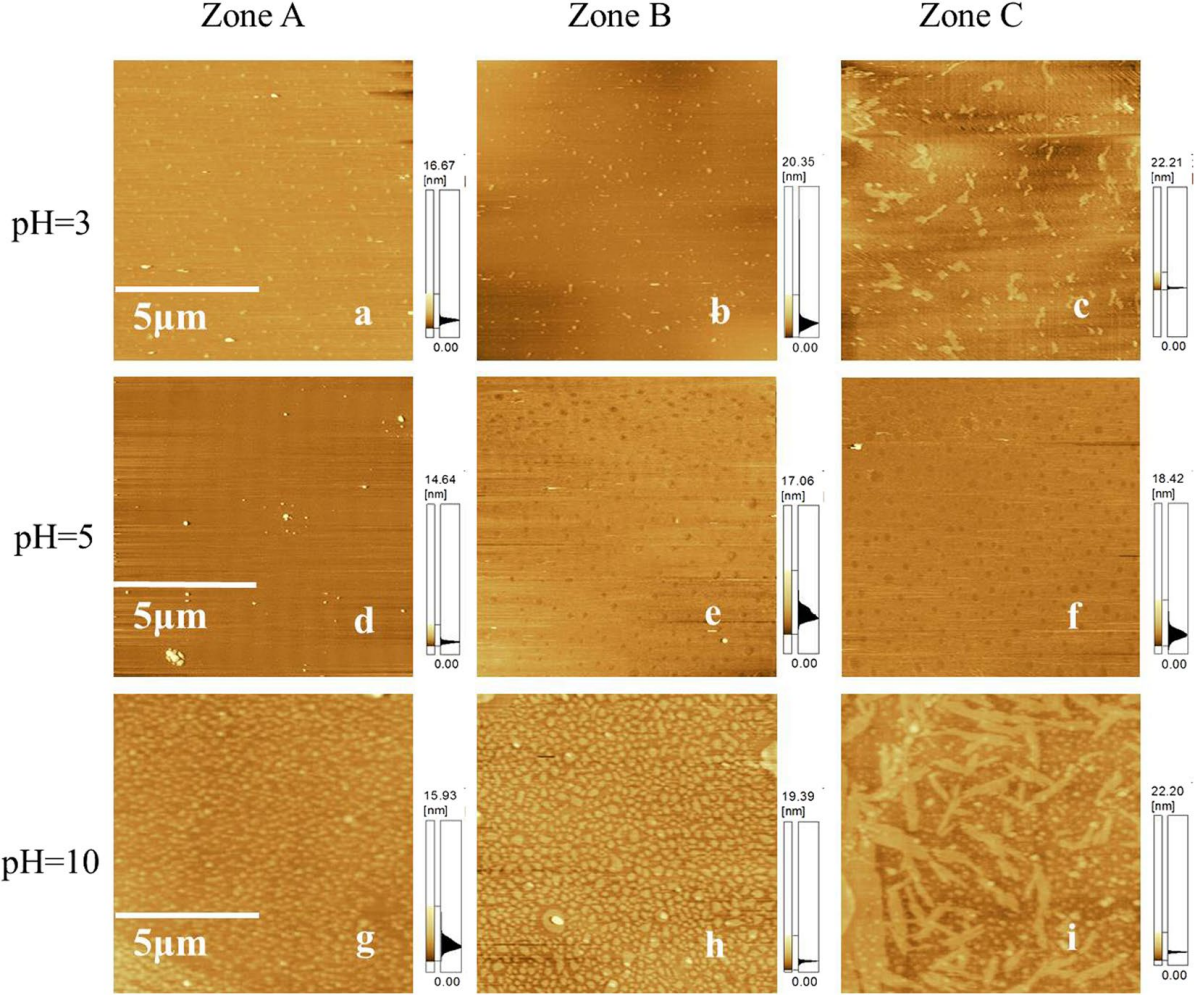
AFM images of DOTAP monolayer on the subphase with different amount of BSA at the surface pressure of 15 mN/m at pH = 3, 5 and 10. ([BSA] = 0 (Zone A); [BSA] = 1 × 10^−8^ M (Zone B); [BSA] = 5 × 10^−8^ M(Zone C)). Scanning range: 10 μm × 10 μm.

At different pHs, the morphologies of DOTAP monolayer on the subphase with different amount of BSA ([BSA] = 0 M, 1 × 10^−8^ M and 5 × 10^−8^ M) at the surface pressure of 15 mN/m are shown in Fig. 9. As can be seen from Fig. 9, at pH = 3, small granular structure appeared in the observed domain of pure DOTAP monolayer Fig. 9(a). When [BSA] = 1 × 10^−8^ M, the amount of granular structure was reduced Fig. 9(b). When [BSA] = 5 × 10^−8^ M, the granular structure disappeared and some complex microdomains appeared in the AFM image Fig. 9(c). At pH = 5, the morphology of pure DOTAP monolayer has the character of tight monolayer structure Fig. 9(d). When [BSA] = 1 × 10^−8^ M and 5 × 10^−8^ M, uniform monolayer structure with defects appeared in the two mixed monolayers Fig. 9(e) and (f). The emergence of defective structure may be caused by the desorption process of BSA. At pH = 10, pure DOTAP monolayer appeared larger granular structure Fig. 9(g). In the system, the desorption process of BSA made DOTAP monolayer form more complex structure Fig. 9(h). When [BSA] = 5 × 10^−8^ M, strip structure and granular structure coexisted in the observed image, which meant more complex structure formed in the desorption process Fig. 9(i). These indicated that the morphologies of mixed DOTAP-BSA monolayers changed a lot because of the desorption process of BSA. Figures 7 and 9 showed that: BSA adsorbed onto DOTAP monolayer firstly and then desorbed from the lipid monolayer as time goes by. The result was consistent with the results obtained from *π* − *A* isotherms and *π* − *t* curves.

## Materials and Methods

### Materials

Crystallized and freeze-dried bovine serum albumin (BSA ≥ 99%) and cationic 1, 2-dioleoyl-3-trimethylammonium-propane (DOTAP) were purchased from Sigma-Aldrich Chemical Company. Anhydrous ethanol, chloroform, hydrochloric acid (HCl), sodium hydroxide (NaOH) and other chemicals were analytically pure and purchased from Tianjin Chemical Company (China). All of them were used without further purification. The phosphate buffer solution (PBS) was used as the subphase. HCl and NaOH were used to adjust the subphase’s pH value. DOTAP was dissolved in a mixture of chloroform and methanol (3: 1, v/v) and the concentration was 1 mg/mL. In order to avoid any measurable surface pressure in the absence of phospholipid molecules, the final concentration of BSA in subphase was very small^35^. The final concentrations of BSA were 5 × 10^−9^ M, 1 × 10^−8^ M, 5 × 10^−8^ M and 8 × 10^−8^ M. Three distilled water were used in the experiment.

## Methods

### Surface pressure measurements

In the work, surface pressure isotherms were measured by a computer-controlled commercial device (Minitrough, KSV, Helsinki, Finland). Lipid monolayers were spread onto the surface of the subphase with different concentrations of BSA using a Hamilton microsyringe. After 15 min of evaporating the organic solutions and equilibrating the monolayer, symmetric compression was performed with the two moving barriers at a constant speed of 10 mm/min. Then, the surface pressures were measured by the Wilhelmy plate technique, and the experimental data were simultaneously recorded by computer. The trough was washed with anhydrous ethanol and rinsed thoroughly with deionized water. Every experimental data was repeated at least three times to obtain good reproducibility. All measurements were carried out at the room temperature (293 ± 1 *K*).

### Surface pressure-time (*π* − *t*) curves measurements

The *π* − *t* curve is used to analyze the penetration kinetics. The measurement of *π* − *t* curve was performed as follows: DOTAP molecules were spread onto the PBS subphase with the absence and presence of BSA ([BSA] = 0 M, 1 × 10^−8^ M). After 30 min of evaporating the organic solutions and equilibrating the monolayer, symmetric compression was performed with two moving barriers at a constant speed of 10 mm/min. After the surface pressure of monolayers reached the certain value of 15 mN/m, interrupted the compression. The change of surface pressure at a constant monolayer area was recorded as a function of time. Every experimental data was repeated at least three times to obtain good reproducibility. All measurements were carried out at the room temperature (293 ± 1 *K*).

### Atomic force microscopy (AFM) observation

Pure DOTAP monolayer and mixed DOTAP-BSA monolayer were transferred onto freshly cleaved micas at the surface pressures of 25 mN/m (after 30 minutes) and 15 mN/m (after two hours), respectively, with a vertical pulling method^36^. The surface morphology feature of deposited monolayer was directly visualized by using an SPM-9500-J3 AFM (Shimadzu Corporation, Japan) in the contact mode. The images (512 × 512 points) in height mode were collected in air at a scanning rate of 1.0 Hz using a Micro-V-shaped Cantilever probe (Olympus Corporation, Japan). The nominal spring constant of the probe was 0.06 N/m. All measurements were carried out at the room temperature (293 ± 1 *K*).

## Conclusion

In this work, the interaction of BSA with the cationic DOTAP at the air-buffer interface has been studied by using the LB technique and AFM. The *π* − *A* isotherms showed that even a small concentration of BSA in subphase could obviously change the property of DOTAP monolayer. The amount of BSA adsorbed onto DOTAP monolayer reached a threshold value at a [BSA] of 5 × 10^−8^ M. [BSA] and pH value could affect the compression quality and the phase transition progress of the lipid monolayer. These results revealed that the interaction mechanism between DOTAP and BSA was affected by the subphase’s pH value. When pH = 3 and 5, the adsorption of BSA was dominated by hydrophobic interaction. However, when pH = 10, the association of BSA with DOTAP at the air-buffer interface was dominated by a combination of electrostatic interaction and hydrophobic interaction. And when pH = 10, BSA could be well separated and purified from complex mixtures. The *π* − *t* curves showed that BSA desorbed from the lipid monolayer as time goes on, and the desorption progress of BSA depended on [BSA] and pH value. AFM images reflected that the change of the morphology feature of lipid monolayer were consistent with the results obtained from surface pressure measurements. The study has potential significance in the fields of separation and purification of biomolecules and biosensor.
